# Optimal Extraction of Antioxidants, Flavonoids, and Phenolic Acids from the Leaves of *Apocynum venetum* L. by Response Surface Methodology with Integrated Chemical Profiles and Bioactivity Evaluation

**DOI:** 10.3390/molecules30194006

**Published:** 2025-10-07

**Authors:** Rulan Qin, Jinhang Song, Qiang Wang, Yingli Guan, Chongning Lv

**Affiliations:** 1 School of Pharmacy and Medicine, Tonghua Normal University, Tonghua 134002, China; qrl@thnu.edu.cn (R.Q.); 17543580339@163.com (J.S.); wangqiang@thnu.edu.cn (Q.W.); guanyingli@thnu.edu.cn (Y.G.); 2 School of Traditional Chinese Materia Medica, Shenyang Pharmaceutical University, Shenyang 110006, China

**Keywords:** leaves of *Apocynum venetum* L., flavonoids, phenolic acids, antioxidant activity, UHPLC-DAD-ESI-QTOF/MS, response surface methodology, Pearson correlation analysis

## Abstract

The leaves of *Apocynum venetum* L. (*A. venetum* L.) are a functional food that plays an important role in antioxidation due to its high content of flavonoids and phenolic acids. Therefore, the extraction process of leaves of *A. venetum* L. is closely related to their activity. In this study, ultra-high-performance liquid chromatography (UHPLC) coupled with diode array detector (DAD), electrospray ionization (ESI), and quadrupole time-of-flight mass spectrometry (QTOF/MS) techniques has been established for qualitative and quantitative analysis of three phenolic acids and six flavonoids in the leaves of *A. venetum* L. Ultrasonic-assisted extraction conditions for the maximum recovery of phenolic and flavonoid compounds with a high antioxidation effect were optimized by response surface methodology (RSM). The optimum extraction conditions were as follows: ethanol concentration 64%, extraction time 20 min, and liquid-to-solid ratio 16:1 mL/g. The yields of three phenolic acids and six flavonoids under the optimal process were found to be 8.932 ± 0.091 and 20.530 ± 0.198 mg/g, respectively, which matched with those predicted (8.751 and 20.411 mg/g) within a 95% confidence level. Antioxidant activities based on ABTS and DPPH assays showed that the optimal extracts had strong activities compared with those of conventional reflux extraction methods. Moreover, the contribution of total and individual phenolic acids and flavonoids to antioxidant activity was also estimated by Pearson correlation analysis.

## 1. Introduction

The leaf of *Apocynum venetum* L. (Luobuma in Chinese) is a medicinal and edible botanical resource that possesses numerous effects, such as calming the liver and nerves, heat-clearing, promoting diuresis, etc. Meanwhile, luobuma tea has also been used as a popular beverage prepared from *Apocynum venetum* L. in combination with hot or boiling water in Europe, North America, and East Asia [1]. Modern phytochemical and pharmacological studies have reported that the leaves of *A. venetum* L. are rich in multiple classes of phytochemicals, with flavonoids and phenolic acids being the most prominent class [2,3]. These compounds have been related to the antioxidant [4], cardioprotective [5], neuroprotective [6], anti-inflammatory [7], and antidepressant-like effects [8,9] of the leaves of *A. venetum* L., and the flavonoid hyperoside is the major chemical constituent and commonly considered as its index for quality evaluation [10]. Additionally, the leaves have been certified to contain various minerals, which can be absorbed into human body, such as sodium, magnesium, calcium, iron, manganese, potassium, etc. [11].

The aforementioned well-reported bioactive activities associated with flavonoids and phenolic acids have led to studies on optimization of their extraction technology from the leaves of *A. venetum* L. For example, Zhang [12], Huang [13], Zhou [14], and Shi [15] et al. improved the purification of total flavonoids, tannins, and polyphenols by macroporous resins, respectively. Tan [16] and Lin [17] et al. employed ionic liquid-based ultrasonic-assisted or microwave-assisted extraction for optimum extraction of total flavonoids or the active components hyperin and isoquercitrin. Wang [18], Ran [19], Bai [20], and Wu [21] et al. developed reflux, microwave, ultrasound-assisted, and ultrasonic-assisted enzymatic hydrolysis or flash extraction technology for optimum flavonoid extraction using orthogonal array design (OAD) or response surface methodology (RSM). Turahun et al. optimized the reflux extraction conditions for polyphenols by OAD [22]. Additionally, the extraction of potential anti-diabetic components of *Apocynum venetum* L. and polysaccharides based on reflux or double-enzyme methods has been optimized by employing RSM [23,24].

The purpose of the present work was to optimize extraction parameters (ethanol concentration, ultrasonic time, and the ratio of solvent to solid) based on the content of flavonoids and phenolic acids from the leaves of *A. venetum* L. by response surface methodology (RSM). The extracts corresponding to the optimal conditions were fully characterized in terms of individual flavonoids and phenolic acids performed by UHPLC-DAD-ESI/MS analysis. The antioxidant assay was undertaken for each investigated extraction condition of the extracts by DPPH and ABTS assays. Moreover, correlations between levels of the major chemical constituents and their antioxidant activities were also investigated by Pearson correlation analysis. This research may provide a theoretical basis for further development and utilization of the leaves of *A. venetum* L.

## 2. Results and Discussion

### 2.1. Phytochemical Profile of the Leaves of A. venetum L. by UHPLC-QTOF-MS

**Figure 1:**
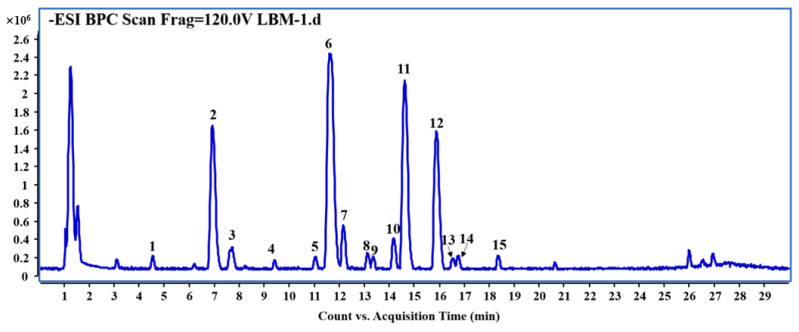
Representative base peak chromatogram (BPC) of leaves of *A. venetum* L. in the negative ion mode. See Table 1 for the peak numbers and see Section 3.5, Qualitative and Quantitative Analysis of Phytochemicals by UHPLC-DAD-QTOF-MS, for UHPLC-QTOF-MS conditions.

**Figure 2:**
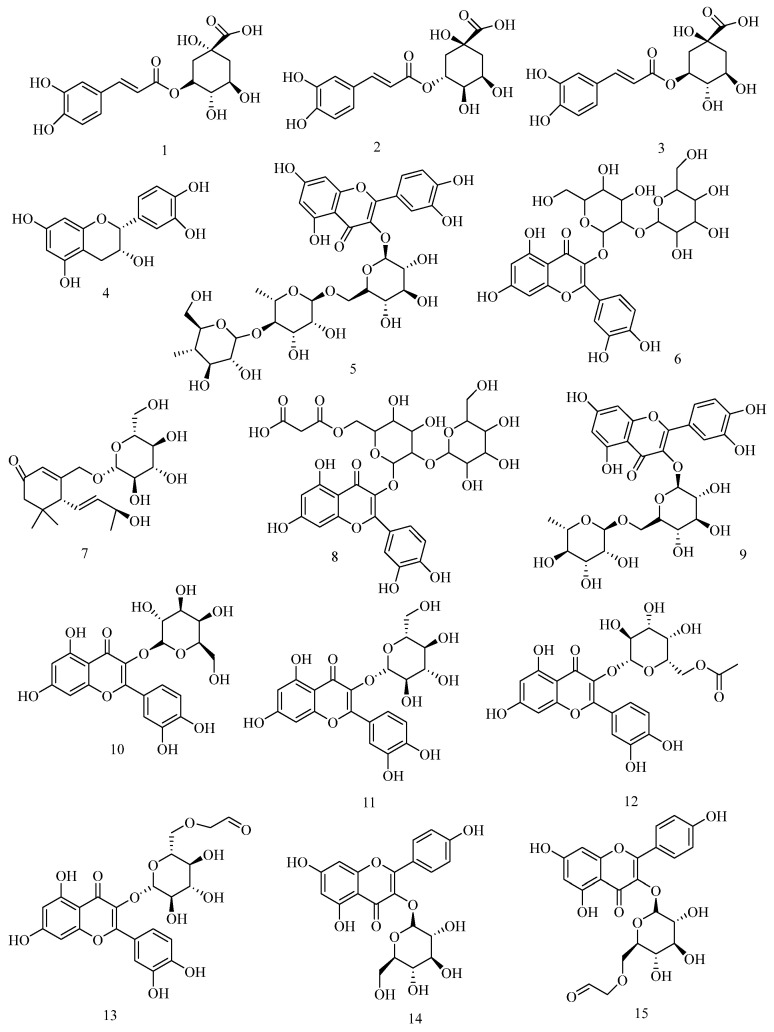
The structures of 15 compounds were identified by UHPLC-DAD-QTOF-MS.

To conduct a comprehensive analysis for the leaves of *A. venetum* L. and lay the foundation for optimization of the extraction process, a 65% methanol extract of *A. venetum* L. leaves was subjected to an UHPLC-ESI-QTOF-MSn analysis. As a result, a total of 15 compounds, including 10 flavonoids, four phenolic acids, and one ionone glucoside, were identified or tentatively characterized (Figure 1 and Figure 2, Table 1). Among them, six unequivocal peaks were unambiguously assigned as chlorogenic acid (peak 2), baimaside (peak 6), rutin (peak 9), hyperoside (peak 10), isoquercetin (peak 11), and astragalin (peak 14) by direct comparison of their retention time and MS spectra with reference compounds, respectively. Because of the absence of reference compounds, other peaks were deduced by high-accuracy quasi-molecular ions such as [M−H]^−^, [2M−H]^−^, [M+Cl]^−^, and [M+HCOO]^−^ within a mass error of 5.0 ppm and fragmentation patterns combined with the previous phytochemical literature.

### 2.2. Model Adequacy

The effect of ethanol concentration, extraction time, and liquid-to-solid ratio on the extraction efficiency of the nine selected representative compounds from two categories of flavonoids and phenolic compounds, measured by content, are summarized in Table 2. The results for yield of total flavonoids and phenolic acids (T_F_ and T_P_) as well as antioxidant activities (IC_50_ values for the ABTS and DPPH assays) were selected as the response variables. The adequacy of the model was investigated by the coefficient of regression (R^2^) and the significance of the coefficients of the model was verified with analysis of variance (ANOVA) by computing the F-values at 0.001, 0.01, and 0.05 probability.

As shown in Table 3, F values (20.54–33.20) obtained from the models were statistically significant (*p* < 0.01), while that of the lack-of-fit was not significant (*p* > 0.05), which indicated that the developed model adequately explains the relationship between the responses and independent variables. Meanwhile, the quadratic polynomial equations for the relation of the test variables (X) and response variables (Y) about total phenolic acids, total flavonoids, and IC_50_ values for the ABTS and DPPH assays were obtained by multivariate regression analysis of experimental data. Each quadratic polynomial equation is listed as follows: Y = 8.66 + 0.16X_1_ + 0.82X_2_ + 0.60X_3_ − 0.45X_1_X_2_ − 0.043X_1_X_3_ − 0.17X_2_X_3_ − 0.19X_1_^2^ − 1.32X_2_^2^ − 1.31X_3_^2^, Y = 20.46 + 0.76X_1_ + 0.38X_2_ + 0.64X_3_ − 0.50X_1_X_2_ − 0.23X_1_X_3_ − 0.034X_2_X_3_ − 1.13X_1_^2^ − 3.99X_2_^2^ − 3.63X_3_^2^, Y = 0.037 − 1.85 × 10^−3^X_1_ − 1.500 × 10^−3^X_2_ − 3.125 × 10^−3^X_3_+ 4.500 × 10^−3^X_1_X_2_ − 1.250 × 10^−3^X_1_X_3_ − 0.011X_2_X_3_−7.500 × 10^−5^X_1_^2^ + 0.022X_2_^2^ + 6.425 × 10^−3^X_3_^2^, and Y = 0.22 − 0.033X_1_ − 0.027X_2_ − 0.023X_3_ + 0.026X_1_X_2_ − 0.024X_1_X_3_ + 1.000 × 10^−3^X_2_X_3_ + 4.325 × 10^−3^X_1_^2^ +0.14X_2_^2^ + 0.12X_3_^2^.

Furthermore, the coefficient of determination (R^2^) and adjusted coefficient of determination (R^2^ adj) values of all four responses exceeded 0.9166, which suggested a good accuracy of the model and conformity of the experimental and predicted values. The generated response surface 3D graphs corresponding to each response by using Design-Expert.V8.0.6 software are shown in Figure 2.

**Figure 3:**
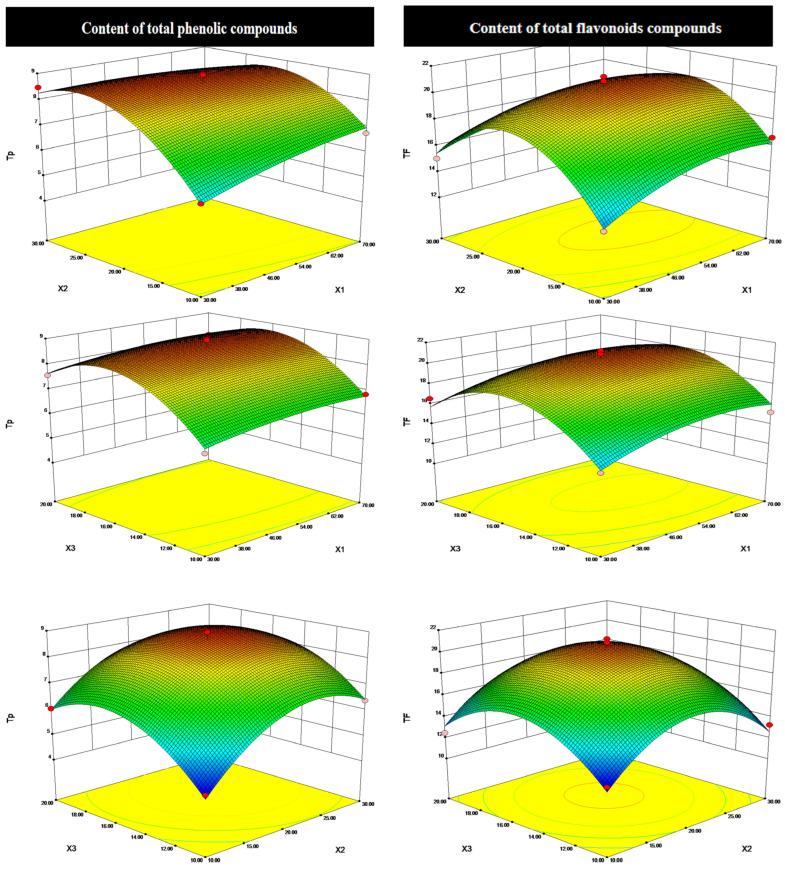

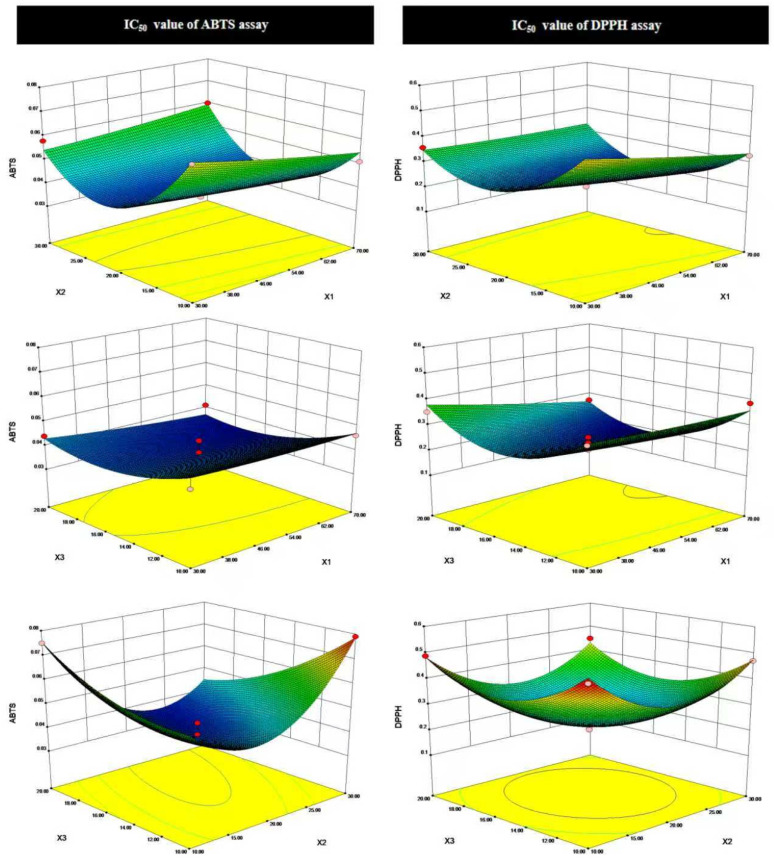
Interactive effect of different variables (X_1_: ethanol concentration, %; X_2_: extraction time, min; X_3_: liquid-to-solid ratio, mL/g) on the total phenolic acids (T_P_), total flavonoids (T_F_), and ABTS and DPPH radical scavenging activity, respectively.

As shown in Figure 3 and Table 3, the *p*-value of each model term indicated that the quadratic terms of X_2_^2^ and X_3_^2^ have highly significant effects with T_P_ and T_F_ (*p* < 0.001), which indicated the important role of extraction time and liquid-to-solid ratio on content. T_F_ and T_P_ increased from 10 to 20 min and 10 to 16 mL/g, while gradually declined with the increment of time and liquid-to-solid ratio. In addition, it can be easily seen that the linear terms of X_2_ and X_3_ and the interaction term of X_1_X_2_ were also highly significant terms (*p* < 0.05) for T_F_, and the significant effect of X_1_ and X_1_^2^ on T_F_ implies the effect of ethanol concentration; however, the other terms’ effects were not significant (*p* > 0.05). Tan et al. [16] also observed an increase first and then a decreased trend in the extraction of flavonoids by optimizing the liquid-to-solid ratio and temperature using the ultrasound-assisted extraction on leaves of *A. venetum* L., obtaining an optimum value of 22 mL/g and 19 min. A similar trend was also reported by Wang and Wu et al. [18,21], who studied the influence of the percentage of ethanol, time, and liquid-to-solid ratio on the extraction of total flavonoids from leaves of *A. venetum* L.

As for antioxidant activity, it is clearly observed how a variance in the extraction time (X_2_) significantly changed the antioxidant capacity of the samples for both the ABTS and DPPH assays (*p* < 0.001). Further, either the quadratic terms of X_3_^2^ or the interaction term of X_1_X_2_ influenced the antioxidant capacity based on the ABTS assay (*p* < 0.01 and *p* < 0.05, respectively). Interestingly, the authors also obtained an important role of the quadratic terms of X_3_^2^ (*p* < 0.001) and linear term of X_1_, X_2_, and X_3_ (*p* < 0.05) by the DPPH assay. Jin et al. [25] also obtained a differential coefficient in the antioxidant capacity (ABTS and DPPH assays) of flavonoid compounds from alfalfa when they used identical extraction parameters. Moreover, the study also indicated that the maximum scavenging capacity of ABTS and DPPH radicals appeared when the extraction time reached 60 min and 45 min, but decreased when these values were higher. Similarly, Edirs et al. [26] also obtained that the most influencing extraction factors on antioxidant capacity (measured using the ABTS assay) were temperature and sample-to-solvent ratio; the latter could be attributed to the consistency with the content of antioxidants in the materials, while the former that has not been measured in the present experiment should be worth considering for the follow-up study.

### 2.3. Optimization and Validation of the Extraction Conditions

Based on the above regression analysis and 3D response surface software, the optimum conditions for the maximum T_P_ and T_F_ using comprehensive evaluation values were obtained with an ethanol concentration of 51.41% and 56.40%, extraction time of 22.85 and 20.27 min, and liquid-to-solid ratio of 16.04 and 15.39 mL/g, respectively. For the antioxidant capacity, the optimum minimum IC_50_ values for ABTS and DPPH assays were obtained with an ethanol concentration of 70.00% and 70.00%, extraction time of 20.28 and 20.04 min, and liquid-to-solid ratio of 16.83 and 15.99 mL/g, respectively. Based on a comprehensive consideration of the active component content and activity, predicted values of T_P_ and T_F_ were 8.751 and 20.411 mg/g, and that of the ABTS and DPPH assays were 0.036 and 0.200 mg/mL under the optimum conditions of ethanol concentration 64.09%, extraction time 20.42 min, and liquid-to-solid ratio 15.82 mL/g, respectively.

Considering practical operations, verification experiments designed to verify the suitability of the model equation were conducted under the modified parameters as follows: ethanol concentration, 64%; extraction time, 20 min; liquid-to-solid ratio, 16 mL/g. All experiments under the optimized conditions were performed in triplicate, which resulted in a T_P_ and T_F_ content of 8.932 ± 0.091 and 20.530 ± 0.198 mg/g and IC_50_ of 0.034 ± 0.001 and 0.203 ± 0.009 mg/mL, respectively. The results were close to the predicted value and are displayed in Table 2. Therefore, the model based on integrated chemical and pharmacological evaluation was suitable for the optimization of extraction process of phenolic acids and flavonoids from the leaves of *A. venetum* L.

### 2.4. Comparison of Present Method with Other Reported Methods

To estimate the efficiency of ultrasound on the extraction of antioxidants from the leaves of *A. venetum* L., a comparison was made between the optimal ultrasound method and conventional refluxing extraction technique. The results in Table 2 showed that the content and antioxidant activities obtained by ultrasound were significantly higher than those from using refluxing extraction. This enhancement could be attributed to the increased pores of the cell wall caused by swelling and hydration processes during the ultrasound process, which improve the diffusion and mass transfer [27]. Therefore, the ultrasound-assisted extraction method was a more efficient extraction method in the extraction of antioxidants from the leaves of *A. venetum* L., with a higher yield, shorter time, and less solvent consumption.

### 2.5. Contribution of Phenolic Acids and Flavonoids to Antioxidant Activity

In order to estimate the contribution of chemical composition on antioxidant activity, correlations between levels of the total and individual phenolic acids and flavonoids and antioxidant capacity determined by ABTS and DPPH methods were calculated using Pearson correlation coefficients (r). As shown in Table 4, there was a significant negative correlation between the levels of T_P_ and T_F_ and antioxidant activity measured using the DPPH assay (r = −0.879 and −0.956, respectively), while a weaker one between T_P_ and T_F_ and ABTS radical scavenging activity (r = −0.686 and −0.771, respectively) was presented. Although the trends of both are consistent, a major reason for the observed phenomenon above was that there were significant differences in the interaction between the chemical compositions and different types of radicals. Similar correlations between T_P_ and T_F_ and ABTS and DPPH have been established in various plants [28]. Meanwhile, as Pearson correlation coefficients revealed, chlorogenic acid (**2**), cryptochlorogenic acid (**3**), baimaside (**6**), isoquercetin (**11**), and acetylated hyperoside (**12**) have been proved to be important bioactive ingredients due to their obvious variability with a *p* value of <0.01, and the correlation was also consistent with the content in medicinal materials, which showed a certain regularity.

In addition, astragalin (**14**) deserves our attention. A lower correlation of −0.452 was found for astragalin (**14**) from the ABTS assay; however, higher value of correlation coefficient (−0.704) for it was observed from the DPPH assay. That might be caused by reciprocal interactions, both antagonistic and synergic.

## 3. Materials and Methods

### 3.1. Materials, Chemicals and Reagents

The leaves *A. venetum* L. was collected/harvested from the herb garden of Shenyang Pharmaceutical University in 2023 and authenticated by Prof. Jincai Lu (Department of Chinese medicine, Shenyang Pharmaceutical University). A voucher specimen has been deposited in the authors’ laboratory. The leaves of *A. venetum* L. were dried to constant weight at 40 °C, ground, and subsequently passed through a 60-mesh sieve.

The reference compounds chlorogenic acid (**2**), baimaside (**6**), rutin (**9**), hyperoside (**10**), isoquercetin (**11**), and astragalin (**14**) were purchased from Shanghai Yuanye Bio-Technology Co., Ltd. (Shanghai, China). The purity of each reference standard was determined to be greater than 98% by HPLC analysis.

Acetonitrile (Merck, Darmstadt, Germany), phosphoric acid, and formic acid (Fisher Scientific, Fairlawn, NJ, USA) were of LC-MS grade. Ultra-pure water (18.2 MΩ) was purified daily using a Milli-Q water purification system (Millipore, Milford, MA, USA). All other chemicals and solvents of analytical grade were obtained from Tianjin Concord Technology Co., Ltd. (Tianjin, China).

### 3.2. Preparation of Standard Solution

Primary stock solutions of chlorogenic acid (**2**), baimaside (**6**), rutin (**9**), hyperoside (**10**), isoquercetin (**11**), and astragalin (**14**) were prepared separately at a final concentration of 0.22, 0.21, 0.12, 0.12, 0.20, and 0.13 mg/mL, respectively, which were accurately weighed and dissolved with methanol. A series of working standard solutions was prepared by successive dilution of the stock solution with methanol to generate the calibration standards. All solutions were stored at 4 °C in a refrigerator and equilibrated to room temperature prior to analysis.

### 3.3. Preparation of Samples Solution

#### 3.3.1. Conventional Refluxing Extraction

First, 50 mL of MeOH/H_2_O (50:50) was mixed with 0.5 g of powdered leaves of *A. venetum* L. and then refluxed at 70 °C for 30 min. The lost weight was compensated for after cooling to room temperature and then the sample was filtered through a 0.22 μm filter prior to HPLC analysis. The experiment was performed in triplicate.

#### 3.3.2. Ultrasound Extraction

The extraction from the leaves of *A. venetum* L. was performed by ultrasonic cleaner (KQ-250E) from Kunshan Ultrasonic Instrument Co., Ltd. (Jiangsu, China). First, 0.5 g of powdered leaves was accurately weighted and extracted with ultrasonic assistance with different variant conditions: ethanol concentration (30%, 50%, and 70%), extraction time (10, 20, and 30 min), and the ratio of liquid to raw material (10, 15, and 20 mL/g). Following extraction, the extract solutions were centrifuged at 3000 rpm for 10 min at 4 °C and the supernatant was filtered through a 0.22 μm filter. These experiments were performed in triplicate. One variable was studied in each experiment while the other factors were kept constant. Major influence factors and their levels were obtained and applied in the RSM design.

### 3.4. Optimization of Processing Method by Box–Behnken Design (BBD)

Box–Behnken design (BBD) was conducted using Design-Expert software (version 8.0.6) to optimize the extraction of total flavonoids, phenolic compounds, and antioxidant activities from the leaves of *A. venetum* L. A three-factor, three-level Box–Behnken design which consisted of 17 trials in random order was employed in triplicate with five repetitions at the central point. The three independent variables investigated in the RSM were ethanol concentration (X_1_), extraction time (X_2_), and the ratio of liquid to raw material (X_3_). The whole experimental design matrix is shown in Table 2. A second-order polynomial model was postulated to describe the extraction procedure by the following equation:

$$Y=\beta_{0}+\sum_{i=1}^{n}\beta_{i}{Χ}_{i}+\sum_{i=1}^{n}\beta_{ii}{Χ}_{i}^{2}+\sum_{1<i<j}^{n}\beta_{ij}{Χ}_{i}{Χ}_{j}$$

where Y is the dependent variable, *X_i_*, *X_ii_*, and *X_j_* are independent variables and n is the number of variables (*n* = 3). *β*_0_ is the constant coefficient, *β_i_*, *β_ii_*, and *β_ij_* are the regression coefficients for the linear, quadratic, and interaction terms, respectively.

### 3.5. Qualitative and Quantitative Analysis of Phytochemicals by UHPLC-DAD-QTOF-MS

Analysis of the chemical profile of leaves of *A. venetum* L. was performed on an Agilent 1290 LC instrument equipped with an Agilent G7117B diode array detector and Agilent 6530b QTOF-MS (Agilent Technologies, Santa Clara, CA, USA). An ACQUITY UPLC HSS C_18_ column (100 × 2.1 mm i.d., 1.8 μm, Waters, MA, USA) was used and the mobile phase consisted of 0.1% aqueous formic acid (A) and acetonitrile (B) using the following gradient elution program: 5–20% (B) for 0–15 min, 20–30% (B) for 15–20 min, 30–99% (B) for 20–25 min, and 99–5% (B) for 25–30 min. The column temperature was maintained at 40 °C. The flow rate was 0.3 mL/min and the injection volume was 5 μL. The spectra were recorded in the range of 200–400 nm. Mass detection was operated in negative mode with a scan range of *m*/*z* 100 to 1000 and the key parameters for the mass spectrometric analysis were as follows: the nebulizer gas (nitrogen) pressure was 35 psi and it was used at 325 °C with the flow rate of 10.0 L/min. Meanwhile, the sheath gas (nitrogen) temperature was 350 °C and the flow rate was 12.0 L/min. Additional parameters were defined as listed below: capillary voltage, −3.5 kV; nozzle voltage, −1000 V; fragmentor voltage, 120 V; skimmer voltage, 65 V; Octopole RF, 750 V. All data were acquired and processed by MassHunter workstation software version B.06.00 (Agilent Technologies, Santa Clara, CA, USA).

The contents of phenolic and flavonoid compounds were quantified using the Essentia LC-16 HPLC system equipped with a CLASS-VP workstation (Shimadzu, Kyoto, Japan). Ten μL of the sample extracts was subjected to HPLC analysis with a Thermo ODS-2 Hypersil analytical column (250 mm × 4.6 mm, 5 μm, Thermo Fisher Scientific, Waltham, MA, USA) using a linear gradient with water–phosphoric acid (100:0.2, *v*/*v*) as solvent A and acetonitrile as solvent B. The gradient program was carried out as follows: 13–13% B for 0–10 min, 13–15% B for 10–20 min, 15–15% B for 20–40 min, 15–40% B for 40–60 min, and 40–13% B for 60–75 min. The flow rate was 1 mL/min and the column temperature was kept at 30 °C. The detection wavelength was set at 256 nm.

### 3.6. Semi-Quantitative Analysis

Concentrations of chlorogenic acid (**2**), baimaside (**6**), rutin (**9**), hyperoside (**10**), isoquercetin (**11**), and astragalin (**14**) were determined based on their corresponding calibration curves. Due to the unavailability of reference standards for neochlorogenic acid, cryptochlorogenic acid, and acetylated hyperoside, their contents could not be determined quantitatively. A semi-quantitative approach was adopted using the calibration curves of chlorogenic acid or hyperoside, respectively.

As compounds with analogous chemical structures share similar molar absorptivity (*ε*) at a specific detection wavelength, the UV spectra of the neochlorogenic acid (**1**) and cryptochlorogenic acid (**3**) were identical to that of chlorogenic acid (**2**) across the 200–300 nm range, in which the characteristic absorption maximum was at 244 nm [29], and that of acetylated hyperoside (kaempferol 3-O-(6″-*O*-acetyl) glucoside) (**12**) was also identical to hyperoside (**10**). Accordingly, the contents of neochlorogenic acid (**1**) and cryptochlorogenic acid (**3**) were quantified based on the calibration of chlorogenic acid (**2**), while the acetylated hyperoside (kaempferol 3-O-(6″-*O*-acetyl) glucoside) (**12**) was determined according to the calibration of hyperoside (**10**). A similar assumption was applied by Rüfer et al. [30]. Taking neochlorogenic acid (NCA, **1**), for example, its concentration was calculated by chlorogenic acid (CA, **2**) using Equations (1) and (2):

(1) $$C_{NCA}=\frac{kA_{NCA}-b}{a}$$

(2) $$k=\frac{E_{CA}}{E_{NCA}}=\frac{\varepsilon_{CA}/M_{CA}}{\varepsilon_{NCA}/M_{NCA}}=\frac{M_{NCA}}{M_{CA}}$$

where *C_NCA_* and *A_NCA_* represent the concentration and peak area of NCA, *a* and *b* are the slope and intercept of the calibration curve of CA, respectively, *k* is the conversion factor of the peak area of NCA relative to peak area of CA, and *E_NCA_* and *E_CA_* are the mass absorptivities of NCA and CA, respectively. *ε_NCA_* and *ε_CA_* are the corresponding molar absorptivities of NCA and CA, and *M_NCA_* and *M_CA_* are the molecular weights of NCA and CA.

### 3.7. Determination of Antioxidant Activities

The antioxidant activities of the extracts were evaluated by 2,2′-azino-bis (3-ethylbenzthiazoline-6-sulfonic acid) (ABTS) and 2,2-diphenyl-1-picrylhydrazyl (DPPH) radical scavenging activity assays according to the method displayed by Ossowski and Yang et al. [31,32], with minor modifications. In both assays, Vitamin C was used as a positive control and absolute alcohol was employed as the blank control. The radical scavenging activity, expressed as inhibition rate (IR), was calculated using the following equation:

IR (%) = (A_0_ − A_s_)/A_0_ × 100%

where A_s_ and A_0_ represent the absorbance of the ABTS/DPPH radical solution with or without the sample, respectively. The corresponding half-maximal inhibitory concentration (IC_50_) was derived from the concentration–response curve by nonlinear regression analysis using the GraphPad Prism 5.0 software. All determinations were performed in triplicate.

#### 3.7.1. ABTS Assay

The ABTS^•+^ working solution was prepared by mixing equal volumes of 7 mM ABTS solution and 2.45 mM potassium persulfate solution. This solution was incubated for 16 h at room temperature in the dark and diluted with methanol until reaching an absorbance of 0.7 ± 0.05 at 734 nm observed using a UV-vis spectrophotometer (TU-1901, Purkinje General Instrument Co., Ltd., Beijing, China). The assay consisted in the addition of 0.1 mL of a series of sample dilutions to 2.7 mL of ABTS^•+^ working solution and measuring the absorbance at 734 nm after 10 min of incubation.

#### 3.7.2. DPPH Assay

In brief, 0.1 mL of a series of sample dilutions was mixed with 1.2 mL of DPPH solution (0.02 mM, dissolved in absolute alcohol) and incubated in the dark at room temperature for 30 min. The absorbance of the resulting mixtures was measured at 517 nm.

### 3.8. Verification of the Model

The extraction conditions were optimized using response surface methodology (RSM) to maximize the yields of total phenolic acids and total flavonoids (T_F_), as well as the antioxidant activities (ABTS and DPPH radical scavenging). The corresponding response values were determined under the optimum extraction conditions. Finally, the experimental data were compared with the predicted values based on the standard errors to verify the reproducibility and robustness of the model.

### 3.9. Correlations Between Levels of the Major Chemical Constituents and Their Antioxidant Activities

In order to achieve a high sight of the relationships among the total phenolic, the total flavonoids, and the analyzed compounds and antioxidant capacities, a correlation analysis was conducted. Pearson correlation analysis was performed using SPSS 17.0 for Windows (SPSS, Inc., Chicago, IL, USA). One-way analysis of variance (ANOVA) was used to test the hypotheses at the 0.05 significance level.

## 4. Conclusions

In the present study, a comprehensive strategy that integrated chemical profiles and bioactivity evaluation was firstly established for improving the combination of three phenolic acids, six flavonoids, and antioxidant activities from the leaves of *A*. *venetum* L. With the integrated quantitative and bioactivity index, the extraction conditions, including three factors (ethanol concentration, ultrasonic time, and the ratio of liquid to solid), were optimized and obtained by the aid of BBD and RSM. The maximum extraction yields and antioxidant activities were 8.932 ± 0.091 mg/g, 20.530 ± 0.198 mg/g, 0.034 ± 0.001 mg/mL, and 0.203 ± 0.009 mg/mL under the optimum extraction conditions at an ethanol concentration of 64%, ultrasonic time of 20 min, and the ratio of solvent to solid of 16 mL/g, which is close to the predicted yields of 8.751 mg/g, 20.411 mg/g, 0.036 mg/mL, and 0.200 mg/mL, respectively. The comparative studies revealed that the extract yields and antioxidant activities achieved by UAE were superior to those obtained by conventional reflux extraction. Moreover, Pearson correlation analysis revealed that chlorogenic acid (**2**), cryptochlorogenic acid (**3**), baimaside (**6**), isoquercetin (**11**), and acetylated hyperoside (**12**) were important bioactive ingredients in view of the content and radical scavenging activity. In particular, semi-quantitative methods can serve as an effective solution for determining the content of compounds that lack reference standards and have similar chemical structures. For example, in this paper, we employed this approach to determine the content of compound 1 and compound 3. These findings not only facilitate efficient and feasible ultrasonic extraction for the recovery of phenolic acids and flavonoids from the leaves of *A*. *venetum* L. but also pave the way for further exploration of leaves of *A*. *venetum* L. as a homologous medicine-food resource with antioxidant properties.

## Figures and Tables

**Table 1:** Characterization of the chemical constituents from leaves of *A. venetum* L. by UHPLC–QTOF–MS.

Peak	t_R_	Identification	Formula	Quasi-Molecular	Observed	Calculated	ppm	Fragement Ions
No.	(min)			Ion	Mass (Da)	Mass (Da)		
1	4.56	Neochlorogenic acid (NCA)	C_16_H_18_O_9_	[M−H]^−^	353.0889	353.0878	3.12	191.0595 [M−H-C_9_H_6_O_3_]^−^ 179.0384 [M−H-C_7_H_10_O_5_]^−^
2	6.98	Chlorogenic acid (CA)	C_16_H_18_O_9_	[M−H]^−^	353.0893	353.0878	4.25	191.0606 [M−H-C_9_H_6_O_3_]^−^
				[2M−H]^−^	707.1812	707.1829	2.40	179.0376 [M−H-C_7_H_10_O_5_]^−^
3	7.70	Cryptochlorogenic acid (CCA)	C_16_H_18_O_9_	[M−H]^−^	353.0890	353.0878	3.40	191.0594 [M−H-C_9_H_6_O_3_]^−^
								179.0378 [M−H-C_7_H_10_O_5_]^−^
4	9.40	(−)-Epicatechin (EC)	C_15_H_14_O_6_	[M−H]^−^	289.0737	289.0718	6.43	245.0847 [M−H-CO_2_]^−^
5	11.04	Rutin-hexoside (RH)	C_33_H_40_O_21_	[M−H]^−^	771.1969	771.1989	−2.59	609.1435 [M−H-C_6_H_10_O_5_]^−^
								301.0355 [M−H-C_6_H_10_O_5_ -C_12_H_20_O_9_]^−^
6	11.64	Quercetin 3-*O*-β-D-glucosyl-(1→2)-β-D- glucoside (Baimaside, BM)	C_27_H_30_O_17_	[M−H]^−^	625.1416	625.1410	0.96	463.0865 [M−H-C_6_H_10_O_5_]^−^
								301.0351 [M−H-2×C_6_H_10_O_5_]^−^
7	12.18	Apocynoside I (AI)	C_19_H_30_O_8_	[M+Cl]^−^	421.1630	421.1635	−1.19	223.1368 [M−H-C_6_H_10_O_5_]^−^
				[M+HCOO]^−^	431.1919	431.1923	−0.93	205.1264 [M−H-C_6_H_10_O_5_ -H_2_O]^−^
8	13.14	Quercetin 3-*O*-(6‴-*O*-malonyl)-β-D- glucosyl- (1→2)-β-D-glucoside (Malonated baimaside, MBM)	C_30_H_32_O_20_	[M−H]^−^	711.1389	711.1414	−3.52	625.1395 [M−H-C_3_H_2_O_3_]^−^
								463.0868 [M−H-C_3_H_2_O_3_ -C_6_H_10_O_5_]^−^
								301.0346 [M−H-C_3_H_2_O_3_ -2×C_6_H_10_O_5_]^−^ -2×C_6_H_10_O_5_]^−^
9	13.34	Rutin (Ru)	C_27_H_30_O_16_	[M−H]^−^	609.1436	609.1461	−4.10	301.0347 [M−H-C_12_H_20_O_9_]^−^
				[M+Cl]^−^	645.1207	645.1228	−3.26	177.9897 [M−H-C_19_H_27_O_11_]^−^
10	14.20	Hyperoside (Hyp)	C_21_H_20_O_12_	[M−H]^−^	463.0866	463.0882	−3.46	301.0369 [M−H-C_6_H_10_O_5_]^−^
11	14.63	Isoquercetin (Ique)	C_21_H_20_O_12_	[M−H]^−^	463.0883	463.0882	0.22	301.0371 [M−H-C_6_H_10_O_5_]^−^
12	15.89	Quercetin-3-*O*-(6″-*O*-acetyl)-galactoside (Acetylated hyperoside, AHyp)	C_23_H_22_O_13_	[M−H]^−^	505.0979	505.0988	−1.78	463.0866 [M−H-C_2_H_2_O]^−^
								301.0370 [M−H-C_2_H_2_O -C_6_H_10_O_5_]^−^
13	16.52	Quercetin-3-*O*-(6″-*O*-acetyl)-glucoside (Acetylated isoquercetin, AI)	C_23_H_22_O_13_	[M−H]^−^	505.0969	505.0988	−3.76	463.0866 [M−H-C_2_H_2_O]^−^
								301.0365 [M−H-C_2_H_2_O -C_6_H_10_O_5_]^−^
14	16.78	Astragalin	C_21_H_20_O_11_	[M−H]^−^	447.0927	447.0933	−3.76	285.0420 [M−H-C_6_H_10_O_5_]^−^
15	18.38	Kaempferol-3-*O*-(6″-*O*-acetyl)-glucoside (Acetylated astragalin)	C_23_H_22_O_12_	[M−H]^−^	489.1022	489.1038	−3.27	447.0918 [M−H-C_2_H_2_O]^−^
								285.0427 [M−H-C_2_H_2_O-C_6_H_10_O_5_]^−^

**Table 2:** Box–Behnken experimental design for the independent variables, content of phenolic acids and flavonoids in leaves of *A. venetum* L., quantified by HPLC-DAD, and antioxidant activity, determined by ABTS and DPPH method ($\bar{x}$ ± *s*, *n* = 3).

Run	Independent			Phenolic Acids (mg/g)				Flavonoids (mg/g)							IC_50_ (mg/mL)	
	Variables															
	X_1_	X_2_	X_3_	1	2	3	T_p_	6	9	10	11	12	14	T_F_	ABTS	DPPH
1	50	30	20	1.851	4.732	0.551	7.132	6.393	0.174	0.051	3.491	3.163	0.162	13.421	0.040	0.450
				±0.021	±0.081	±0.012	±0.112	±0.172	±0.006	±0.002	±0.040	±0.036	±0.005	±0.171	±0.002	±0.018
2	50	30	10	1.421	4.561	0.402	6.381	6.402	0.153	0.043	3.880	2.642	0.141	13.252	0.079	0.471
				±0.032	±0.054	±0.015	±0.109	±0.068	±0.003	±0.002	±0.042	±0.040	±0.006	±0.162	±0.003	±0.021
3	50	20	15	1.873	6.352	0.773	8.992	9.991	0.172	0.031	6.792	4.030	0.201	21.212	0.035	0.213
				±0.029	±0.067	±0.020	±0.098	±0.110	±0.004	±0.001	±0.062	±0.043	±0.005	±0.218	±0.001	±0.008
4	30	10	15	1.916	3.353	0.478	5.753	6.332	0.123	0.021	4.558	2.421	0.070	13.520	0.067	0.477
				±0.042	±0.058	±0.021	±0.061	±0.081	±0.005	±0.001	±0.053	±0.036	±0.003	±0.180	±0.003	±0.014
5	50	10	20	1.393	4.144	0.501	6.028	6.158	0.149	0.039	3.443	2.532	0.161	12.475	0.075	0.488
				±0.026	±0.047	±0.012	±0.072	±0.065	±0.004	±0.001	±0.049	±0.044	±0.004	±0.152	±0.003	±0.016
6	30	20	10	1.422	4.276	0.491	6.187	7.958	0.132	0.022	3.340	2.258	0.103	13.812	0.044	0.372
				±0.029	±0.041	±0.017	±0.059	±0.119	±0.004	±0.001	±0.056	±0.029	±0.004	±0.165	±0.001	±0.016
7	70	20	10	1.854	4.402	0.592	6.843	7.191	0.131	0.041	4.801	2.880	0.181	15.220	0.046	0.387
				±0.030	±0.043	±0.017	±0.102	±0.132	±0.003	±0.002	±0.071	±0.038	±0.007	±0.180	±0.002	±0.017
8	70	20	20	2.047	5.263	0.738	8.052	8.082	0.193	0.049	4.722	3.822	0.242	17.101	0.041	0.272
				±0.031	±0.062	±0.026	±0.074	±0.090	±0.004	±0.001	±0.101	±0.044	±0.011	±0.203	±0.002	±0.010
9	50	20	15	1.803	5.791	0.751	8.344	9.801	0.152	0.031	6.972	3.751	0.193	20.894	0.038	0.223
				±0.024	±0.066	±0.019	±0.092	±0.110	±0.003	±0.001	±0.071	±0.039	±0.005	±0.218	±0.001	±0.007
10	50	20	15	1.991	5.573	0.833	8.393	9.326	0.139	0.042	6.639	3.993	0.220	20.364	0.043	0.203
				±0.031	±0.059	±0.021	±0.088	±0.096	±0.003	±0.001	±0.067	±0.041	±0.006	±0.199	±0.002	±0.009
11	70	10	15	1.824	4.284	0.642	6.741	7.993	0.181	0.043	5.013	3.232	0.210	16.663	0.051	0.327
				±0.022	±0.054	±0.021	±0.068	±0.113	±0.006	±0.002	±0.094	±0.022	±0.008	±0.170	±0.002	±0.012
12	30	20	20	2.488	4.296	0.782	7.569	8.451	0.092	0.041	5.152	2.701	0.151	16.578	0.044	0.352
				±0.037	±0.051	±0.023	±0.125	±0.082	±0.004	±0.002	±0.106	±0.031	±0.006	±0.150	±0.002	±0.010
13	50	20	15	1.923	5.922	0.779	8.622	8.968	0.174	0.041	6.144	3.962	0.211	19.486	0.037	0.231
				±0.030	±0.062	±0.020	±0.086	±0.101	±0.004	±0.001	±0.065	±0.041	±0.006	±0.196	±0.001	±0.008
14	50	10	10	1.384	2.841	0.381	4.601	6.052	0.153	0.040	3.170	2.674	0.130	12.212	0.062	0.513
				±0.021	±0.031	±0.013	±0.052	±0.116	±0.005	±0.002	±0.031	±0.040	±0.005	±0.163	±0.002	±0.011
15	50	20	15	2.147	5.982	0.818	8.952	9.843	0.172	0.042	6.061	4.030	0.203	20.342	0.035	0.253
				±0.032	±0.055	±0.021	±0.090	±0.101	±0.004	±0.001	±0.061	±0.041	±0.007	±0.175	±0.001	±0.011
16	30	30	15	2.581	5.091	0.802	8.473	8.301	0.091	0.031	3.429	3.082	0.121	15.050	0.058	0.358
				±0.041	±0.059	±0.026	±0.131	±0.096	±0.004	±0.001	±0.044	±0.068	±0.003	±0.162	±0.002	±0.012
17	70	30	15	2.282	4.660	0.731	7.670	7.314	0.208	0.053	4.422	3.911	0.251	16.151	0.060	0.310
				±0.033	±0.057	±0.011	±0.114	±0.101	±0.004	±0.002	±0.050	±0.069	±0.008	±0.170	±0.002	±0.009
Predicted	64.09	20.42	15.82	2.082	5.852	0.824	8.751	9.418	0.181	0.051	6.383	4.140	0.238	20.411	0.036	0.200
Experimental	64	20	16	2.152	5.968	0.811	8.932	9.390	0.172	0.042	6.560	4.161	0.241	20.530	0.034	0.203
				±0.031	±0.061	±0.012	±0.091	±0.103	±0.003	±0.001	±0.069	±0.042	±0.005	±0.198	±0.001	±0.009
Reflux	50	30	100	2.283	4.941	0.314	7.531	6.441	0.133	0.023	5.162	3.252	0.132	15.132	0.049	0.363
				±0.048	±0.092	±0.011	±0.170	±0.121	±0.004	±0.001	±0.098	±0.051	±0.004	±0.260	±0.002	±0.017
Vc															0.005	0.041
															±0.000	±0.002

**Table 3:** Regression coefficients (β), coefficient of determination (R^2^ and Adj. R^2^), and F-test value of the predicted second-order polynomial models for the total flavonoids and phenolic compounds (T_F_ and T_P_) and antioxidant activities (IC_50_ values for DPPH and ABTS assays).

Factor	Coefficient (β)			
	T_p_	T_F_	ABTS	DPPH
Intercept	8.66	20.46	0.037	0.22
Linear				
X_1_	2.56	6.66 *	1.75	12.00 *
X_2_	63.54 ***	1.69	1.12	8.09 *
X_3_	33.90 **	4.76	4.86	5.68 *
Quadratic				
X_1_^2^	1.79	7.81 *	1.47 × 10^−3^	0.11
X_2_^2^	86.73 ***	97.51 ***	123.14 ***	113.01 ***
X_3_^2^	85.71 ***	80.64 ***	10.82 *	79.74 ***
Interaction				
X_1_X_2_	9.70 *	1.45	5.04	3.61
X_1_X_3_	0.088	0.31	0.39	3.13
X_2_X_3_	1.38	6.53 × 10^−3^	32.93 **	5.55 × 10^−3^
R^2^	0.9771	0.9689	0.9635	0.9714
Adj.R^2^	0.9477	0.9288	0.9166	0.9347
F value (model)	33.20 ***	24.19 **	20.54 **	26.43 **
F value (Lack of Fit)	0.76	2.36	1.98	3.30

X_1_: Ethanol concentration (%), X_2_: Extraction time (min), X_3_: Liquid-to-solid ratio (mL/g), R^2^: coefficient of determination. Level of significance: * *p* < 0.05, ** *p* < 0.01, *** *p* < 0.001.

**Table 4:** Correlation coefficients between the levels of total and individual phenolic acids and flavonoids and antioxidant capacity determined by ABTS and DPPH assays.

	Phenolic Acids				Flavonoids							ABTS	DPPH
	(1)	(2)	(3)	T_P_	(6)	(9)	(10)	(11)	(12)	(14)	T_F_		
ABTS	−0.352	−0.672 **	−0.693 **	−0.686 **	−0.804 **	−0.118	−0.048	−0.671 **	−0.629 **	−0.452	−0.771 **	1	0.793 **
DPPH	−0.448	−0.863 **	−0.879 **	−0.879 **	−0.933 **	−0.280	−0.117	−0.850 **	−0.860 **	−0.704 **	−0.956 **	0.793 **	1

Level of significance: ** *p* < 0.01.

## Data Availability

The original contributions presented in this study are included in the article. Further inquiries can be directed to the corresponding author.
